# Real-Time Aural and Visual Feedback for Improving Violin Intonation

**DOI:** 10.3389/fpsyg.2019.00627

**Published:** 2019-04-02

**Authors:** Laurel S. Pardue, Andrew McPherson

**Affiliations:** Augmented Instruments Laboratory, Centre for Digital Music, Queen Mary University of London, Electrical Engineering & Computer Science, London, United Kingdom

**Keywords:** violin, intonation, motor learning, pedagogy, real-time feedback, aural feedback, visual feedback

## Abstract

Playing with correct intonation is one of the major challenges for a string player. A player must learn how to physically reproduce a target pitch, but before that, the player must learn what correct intonation is. This requires audiation- the aural equivalent of visualization- of every note along with self-assessment whether the pitch played matches the target, and if not, what action should be taken to correct it. A challenge for successful learning is that much of it occurs during practice, typically without outside supervision. A student who has not yet learned to hear correct intonation may repeatedly practice out of tune, blithely normalizing bad habits and bad intonation. The real-time reflective nature of intonation and its consistent demand on attention make it a ripe target for technological intervention. Using a violin augmented to combine fingerboard sensors with audio analysis for real-time pitch detection, we examine the efficacy of three methods of real-time feedback for improving intonation and pitch learning. The first, aural feedback in the form of an in-tune guide pitch following the student in real-time, is inspired by the tradition of students playing along with teachers. The second is visual feedback on intonation correctness using an algorithm optimized for use throughout normal practice. The third is a combination of the two methods, simultaneously providing aural and visual feedback. Twelve beginning violinists, including children and adults, were given four *in-situ* 20–30 min lessons. Each lesson used one of the intonation feedback methods, along with a control lesson using no feedback. We collected data on intonation accuracy and conducted interviews on student experience and preference. The results varied by player, with evidence of some players being helped by the feedback methods but also cases where the feedback was distracting and intonation suffered. However interviews suggested a high level of interest and potential in having such tools to help during practice, and results also suggested that it takes time to learn to use the real-time aural and visual feedback. Both methods of feedback demonstrate potential for assisting self-reflection during individual practice.

## 1. Introduction

While the author was sitting in on a group intermediate violin class, Kate Conway, head of London's Suzuki Hub, asked her students what was the most important issue to focus on while playing a piece. To demonstrate, she played a piece twice. The first time she played with good intonation but poor tone and bow technique. The second time she played with excellent tone but poor intonation. She then asked the group of around 15 students which one was worse to listen to. The students quickly agreed the example with poor intonation was clearly less enjoyable.

Learning correct intonation is a major issue for string players. It requires developing a refined internal sense of pitch to recognize correct intonation, the proprioceptive knowledge to physically place a finger close to target, and an internal feedback loop to adjust finger placement to optimize intonation. Two of the practical challenges are being able to determine whether a target note is in tune, and then maintaining sufficient aural attention to constantly listen for identifiable error. During practice, where most learning occurs (Sloboda et al., 1996), there is no teacher to help identify intonation error.

This article presents a study of two real-time feedback methods, one aural, and one visual, aimed at aiding learning of correct intonation during practice. As intonation assessment and motor performance are a constantly iterative process, we wanted to investigate whether real-time feedback could assist a beginner student's intonation. We start with a review of existing violin learning technology and then a violin-centric pedagogical overview on learning intonation. We then discuss our selection of feedback methods, how the study was conducted, including an in-the-wild context, before presenting the results of feedback on performance and participant experience. Next, we provide our personal experiences teaching with the technology, and finally, discuss some of the questions unanswered by our study.

### 1.1. Background

There have been a variety of attempts to use technology to intervene in violin learning. One of the first significant attempts to build a violin practice and pedagogical tool was with iMaestro (Ng K. C. et al., 2007; Ng K. et al., 2007; Ng and Nesi, 2008). iMaestro used a Vicon motion capture system for tracking violin performance. One of the main interactions was a 3D visualization of the player that included bowing trajectories.

Recognizing that demands on visual attention elsewhere may impede the use of visual feedback, iMaestro included sonification for real-time feedback (Larkin et al., 2008). Sonifications were designed both continuously and when specific events happened, for instance when a player's bowing angle exceeded a preset threshold, but results on efficacy were inconclusive.

Similarly, Schoonderwaldt and Wanderley (2007) developed visualizations of bow actions for pedagogical reasons. Amongst his many explorations using motion capture in conjunction with Demoucron's strain gauge (Demoucron et al., 2009), Schoonderwaldt developed two-dimensional visualizations tracking the bow's frog in relation to the violin, bow tilt, and derivable information such as bow velocity.

Subsequently the TELMI project, whose stated goal is to provide practical learning tools for advanced players, has been researching how to extend the above works using video motion capture (Volpe et al., 2017) along with examining what information on bowing can be derived purely from audio analysis (Perez-Carrillo, 2016).

There are also commercial interactive learning systems for working through repertoire and less focused on technique (Purely Violin^1^) and larger on-line music learning networks that are not violin specific like SmartMusic^2^ and PRAISE (Yee-King et al., 2014) that include performance analysis tools that evaluate note accuracy and timing^3^.

Though for bagpipe rather than violin, Menzies has tested promising real-time learning tools based on visual feedback in real-world lessons and practice (Menzies, 2015), while Johnson has done extensive work on learning tools targeted toward violin beginners in the classroom. With Van der Linden, Johnson started by using a jacket from a motion capture suit to track performer posture and then provide vibro-tactile feedback to encourage corrections (van der Linden et al., 2011b). The jacket and more accessible technologies were further tested “in the wild” (van der Linden et al., 2011a; Johnson et al., 2012, 2013) to encourage good basic bow technique and posture based on vibro-tactile and visual feedback. One of the main lessons Johnson found was that the use and response to a given feedback method is often very personal to the user; while one user finds visuals flashing distracting, another finds it necessary to draw attention.

#### 1.1.1. Intonation Learning Tools

There are a number of intonation tools for voice and/or violin that use audio analysis to compare a performance with the score highlighting incorrect pitches: both academic (Lu et al., 2008; Lim and Raphael, 2010; Wang et al., 2012) and commercial (Tra La^4^). Non-real-time systems have the drawback of only being useful for reflective evaluation, and a drawback of all is that they rely on a reference score for ground truth which can hinder practice flexibility. Only Wang et al. (2012) and Tra La offer real-time evaluation of intonation.

One intonation feedback tool taking a non-score based approach is descried in De Sorbier et al. (2012), who used a Kinect to track the violin and fingerboard and then displayed a video of the player overlaid with virtual frets enabling the student to see whether their finger is placed in the correct location. Audio pitch analysis was added to inform arrows on the visualization directing which way the student should move their finger to correct to the nearest note.

#### 1.1.2. Pedagogical Approaches to Intonation

We briefly reflect on pedagogical ideas that underpin traditional learning of correct intonation, the need to learn to differentiate correct intonation, and the means by which a player performs a target pitch. Experienced Suzuki violin teacher Kreitman (1998, p. 19) argues that when teaching young children, two of the earliest teaching tasks are “can you differentiate between notes?” followed by “when they are different, can you describe whether the second pitch is higher or lower?” Refined pitch differentiation is learned. Both Micheyl et al. (2006) and Vurma (2014) found differences in pitch discrimination between non-musicians, keyboardists, and string players who have to correct intonation, along with timbral effects on differentiation.

Further, what is considered acceptably in-tune depends on musical familiarity. Non-musicians and musicians have noticeably different standards for what is acceptable intonation (Warren and Curtis, 2015; Larrouy-Maestri, 2018) and how dramatically poor intonation affects a perception of a performance. Still, even non-musicians find overly poor intonation negatively impacts enjoyment.

String players must learn *audiation*, mentally imaging what music should sound like based on aural memory. Edwin Gordon coined the term as an aural equivalent to visualization (Kreitman, 2010, p. 41). Violinist and teacher Michael Martin states in Reel (2014), “Good intonation comes primarily from inside the player's head. If the player isn't hearing—the word we use is ‘audiating’—good intonation in their mind, it's really not going to come out of the instrument.”

Music pedagogy suggests that one of the best ways to form aural memory is by repeated listening. Shinichi Suzuki placed great emphasis on listening (Suzuki and Suzuki, 1983) with the “three major elements of Suzuki's Mother Tongue are listening, imitation, and repetition” (Kohut, 1985, p. 11). Music pedagog Kohut (1985, p. 61) states, “The quality of our musical conception is directly influenced by the quality of the musical performances we hear.…it is therefore critical that the ‘musical ear’ be programmed with superior musical concepts or images.”

**Figure 1:**
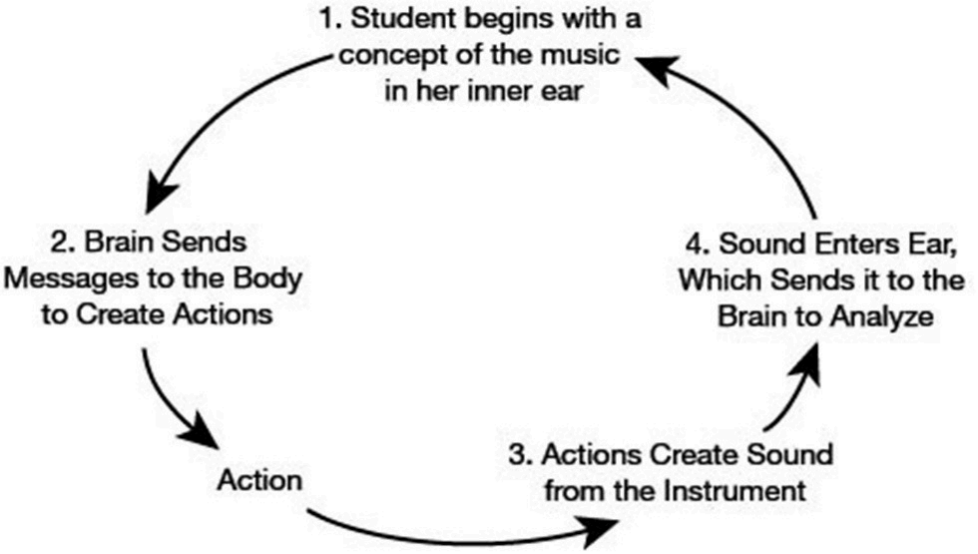
Kreitman's listening loop for performing correctly with good tonalization (redrawn from Kreitman, 2010, p. 43).

Figure 1 depicts a listening loop proposed by Kreitman (2010, p. 43) for tonalization and intonation that is derivative of Adam's closed loop system for motor learning (Adams, 1971; Kempter, 2003, p. 71). For intonation, the listening loop is adjusted so that: the student uses audiation to mentally define the target pitch, thinks about how to physically achieve the target, performs the resultant left-hand action, listens to and evaluates whether the action has resulted in the correct pitch, and then repeats the loop refining the physical action to better match the target till it is reached.

Two major stages in the application of the listening loop toward good intonation are the ability to evaluate whether a pitch is correct, and how to correct it. The listening loop also only works when a student is actively listening. When well-developed, research has found the intonation process becomes automatic and effective beyond conscious attention (Hafke-Dys et al., 2016).

### 1.2. Feedback Methods and Motivations

We are interested in real-world practical tools for assisting violin learning. We developed two methods, one aural and one visual, in order to provide additional technologically driven feedback. We used a custom built augmented violin capable of low-latency pitch detection (Pardue et al., 2015) to evaluate pitch played and used the result to provide intonation feedback. The two types of feedback were:

1. Aural Feedback: low-latency pitch corrected audio of a participant's playing as an aural guide of correctness.
2. Visual Feedback: a low-latency graphic depicting nearest note played and a participant's intonation relative to the note.

In this study we tested both of the above cases, along with a third case combining the previous two, and a control case:

3. Combined Feedback: we provided students with both the aural and visual feedback.
4. No Feedback: this case was intended as a control case to capture intonation performance without an intonation aid.

The aural and visual feedback methods were chosen based on existing analogs in pedagogical practice. Aural feedback was inspired by a common technique in violin lessons where a teacher plays with the student, flexibly acting as an aural guide and providing an example for the student to follow. It is intended both to demonstrate for the student the music as it should be played, reinforcing the student's aural understanding and audiation of the piece, while also serving as a real-time comparison to their own playing to highlight error and encourage correction. Practicing at home by playing along to a recording is widespread (Music Minus One^5^), and there are a handful of commercial options that allow a violinist to practice repertoire with tempo control (ABRSM Scales Trainer^6^, PlayAlong^7^).

A drawback to the use of aural feedback however is that it utilizes a sensory mode that is already required for playing and may be easily ignored, or disruptive (Larkin et al., 2008). In her discussion of how to design learning aids for instruments, Johnson's (2014, p. 48) argues for interactions to use a different modality than those already used in a task, such as visual modes, when not needing a score.

Many modern digital tuners, widely available as software or specialist accessories, analyze pitch played and display the nearest scale note and how far away the performed pitch is. However most commercial tuners are designed for instrument tuning and optimize for accuracy at the cost of speed. Tuners are generally too slow for use in all but the slowest practice (Lim and Raphael, 2010). The idea with our visual feedback method was to design a relatively low-latency high speed version of a digital tuner with a large clear graphic for use at moderate practice tempos.

Another recommendation of Johnson's (2014, p. 126) is that mixed modality can often be best. Mixed modality can combine to emphasize a learning target or a student can refer to one modality in some cases, and another when that first modality is inappropriate. For instance, if a student listening to an aural guide is having trouble deciding whether he/she is sharper or flatter than the guide note, visual feedback may provide simple clarification, or if a student requires a score, visual feedback would be ineffective though aural feedback would remain serviceable.

With these motivations in mind, the accompanying hypotheses for testing are:

1. Hearing an in tune aural guide will enable the student to self-evaluate and correct intonation more easily than playing without any intonation aid.
2. Visual feedback depicting intonation performance will help a student self-evaluate and correct intonation more easily than playing without any intonation aid.
3. Providing both forms of feedback will help a student self-evaluate and correct intonation more easily than playing without any intonation aid. Further, we hypothesize that aural and visual feedback will act in complementary ways yielding the best improvement in intonation.

## 2. Methods

Our primary research aim is to develop practice tools that can eventually be deployed in a home practice session. Though controlled laboratory studies would be more likely to yield easily interpreted results about the efficacy our feedback conditions, repeated interaction studies have found laboratory derived results regularly fail to predict real-world usefulness (Consolvo et al., 2007; Rogers et al., 2007; Jambon and Meillon, 2009; Rogers, 2011; van der Linden et al., 2011a; Kjeldskov and Skov, 2014). Thus, we opted to start by conducting an *in-situ* study, using the context of four real-world lessons and our principal target audience, young beginners, leaving the option to conduct a laboratory study in future.

We conducted lessons with beginner students using an acoustic augmented violin in order to compare the effects of four different intonation feedback methods on intonation execution and perceived helpfulness. Each lesson included study specific repertoire before proceeding to material chosen by the student. At the end of each lesson, we included a brief section related to another study which is not discussed here (Pardue, 2017, p. 243).

The study was conducted using a custom built augmented violin system capable of supporting real-time low-latency pitch correction. Our augmented violin and associated software system is described in detail in Pardue et al. (2015), Pardue (2017). It uses a sensor on the fingerboard to provide a rough hardware based estimate for pitch derived from where the finger contacts the fingerboard. The hardware based estimate is subsequently used to restrict search regions for traditional software pitch estimation techniques such as biased auto-correlation and Yin (De Cheveigné and Kawahara, 2002). The resulting low-latency pitch estimates are then used either with a modern pitch synchronous overlap and add (PSOLA) algorithm to correct the pitch of the violin audio, and/or displayed on a laptop screen as visual feedback. The augmented violin achieves pitch estimation accuracy well in-line with established methods (Pardue et al., 2015; Pardue, 2017).

Study participants played standard acoustic violins we augmented, either half-sized or full-sized as appropriate for the student's height. For cases featuring aural feedback participants were asked to wear one ear of a pair of headphones so that they could hear both their own playing and the pitch corrected audio guide, i.e., audio of the student's performance automatically corrected to the nearest allowed pitch in the selected key. In order to ensure participants were able to differentiate between what they were playing and the pitch corrected audio, we used multi-band compression to alter the sound of the guide.

**Figure 2:**
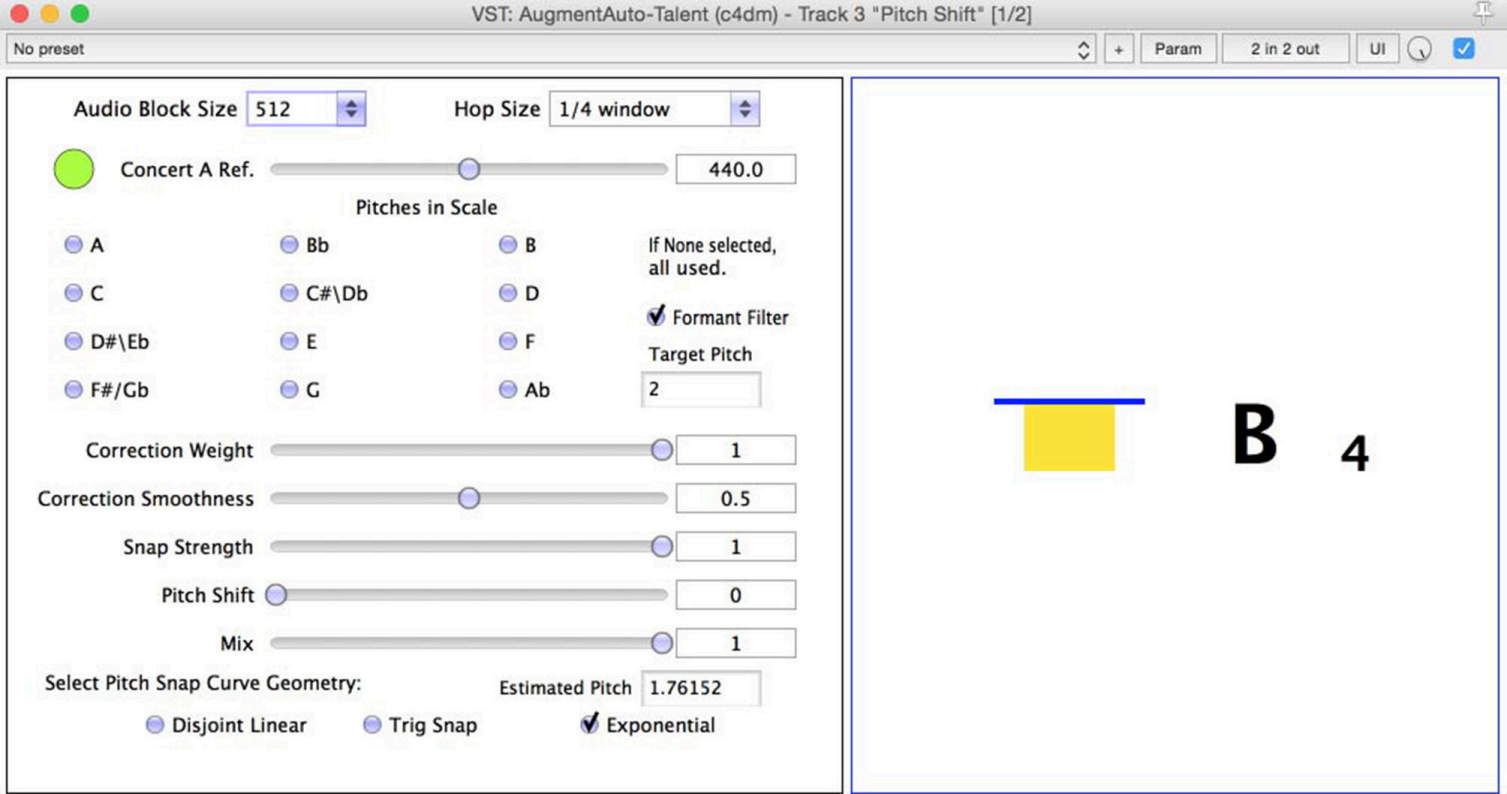
Interface for the selection of pitch snap properties and notes included in the snapped scale **(left)**, along with a display of the performed pitch and the intonation relative to that pitch **(right)**.

Visual feedback was provided as shown in Figure 2 featuring what chromatic note was being played and a colored bar representing whether a student was above or below it. The location, color and size of the error bar represented the direction and level of error. For instance, a small green error bar indicated that the played pitch was close to the note whereas a tall red bar indicated the played pitch was far from the chromatic note. Scores were positioned as near as possible to the screen so that students could switch visual attention between the two easily, however room layout typically meant the two were at least two feet apart.

The combined feedback case simultaneously incorporated both the aural and visual feedback. Participants were allowed to use either form of feedback as they saw fit. The last study case, no feedback, was a control case teaching a lesson as normal but with students playing on the augmented violin. Use of feedback in all lessons was optional. Student participants were not required to look continuously at the visual feedback and were allowed to remove headphones if they found them distracting or uncomfortable.

### 2.1. An “In the Wild” Environment

As most active beginner violin students are children and we asked students to come for four lessons, it was necessary to structure lessons in a way to keep students engaged. This resulted in a continuous compromise between conducting lessons as lessons, and conducting lessons as a study. The lesson context meant the need to challenge students with complicated learning tasks, like new repertoire and technique, while simultaneously responding to study feedback. The drawback of this is that students were not necessarily directing their full concentration toward the aural or visual feedback, but the benefit is that we are able to see real-world interactions and the boundaries of when feedback was or was not useful.

It proved difficult to recruit violin teachers to conduct the lessons, so the first author, who is an experienced violinist, conducted the lessons. Johnson writes extensively about the challenges, benefits, and risks of bias when studying violin practice aids “in the wild” with active researcher participation (Johnson et al., 2012; Johnson's, 2014). She discusses issues such as variation in student concentration, dealing with shy participants, potential areas of, and means for countering bias, and many contextual issues that, though not core to our study, will impact the results. We address some of the potential biases encountered within our study prior to presenting results in section 3.2.1. Van Der Linden and Johnson demonstrate how the many contextual variables faced in “in the wild” studies, and in particular, violin based studies, often yield less definitive results than a similar laboratory study (van der Linden et al., 2011a).

### 2.2. Participants

This study was completed by 12 beginners, two of whom were adults. In order to ensure that students were capable of performing pieces included in the study and have sufficient experience to understand and respond to guidance on pitch, all participants were required to have completed the Associated Board Royal Schools of Music (ABRSM) Grade 1 exam or first half of Suzuki Book 1. We limited our skill level by barring students who might use vibrato. Vibrato is not a beginner technique and was potentially problematic for the study as reliably identifying and interpreting heard vibrato pitch is challenging (Brown and Vaughn, 1996; Geringer et al., 2014; Yang et al., 2016).

Students were offered four free 30 min lessons in return for taking part in the study. Within this paper, different participants are denoted using *Px* where *x* denotes which participant is being referenced. The study was reviewed and approved by the Queen Mary University of London Research Ethics Committee and consent, both written and informed, was obtained from the participants and their parents in case of minors.

Participants were primarily recruited through two sources, London's Suzuki Hub run by Kate Conway, and students taught at a local academy by Sigurd Feiring. One additional adult participant was a professional percussionist with beginner violin skills interested in improving her intonation (P11). Students from the Suzuki Hub were all volunteers, including one parent with beginner level skills (P2). Lessons with Suzuki Hub students were conducted at the Suzuki Hub with parents present. Participants taught by Feiring were selected for an appropriate level of skill and commitment. Due to schooling time constraints, lessons with Feiring's students were targeted for 20 min.

Of 14 initial participants, 11 completed all 4 lessons with 1 student, P7, only failing to complete the control lesson. As P7 experienced and completed interviews on all three different feedback methods, his results are included where applicable giving results for the 12 participants. The included study group aged between 31–33 years (adults) and 8–11 (children), with a mean age of 32 and 9.3 years respectively. Length of prior violin study was between 1 and 7 years (mean 3.7 years) with the grade or Suzuki Book ranging between 1 and 4 (mean 2.2). Half the participants were at a level equivalent to Grade 1. 8 of the 12 participants were female, including one adult and the study group included one autistic child.

#### 2.2.1. Suzuki vs. Traditional Teaching

It was apparent that there were differences between Suzuki Hub and non-Suzuki students (8 and 4 respectively) that might impact on how they responded to the different types of feedback. Suzuki students are taught with an emphasis on aural learning and despite excellent skills otherwise, even the most advanced Suzuki students in the study had difficulty reading music.

Further, the author/teacher found that for any piece a Suzuki student was not confident with, it was implicitly expected that the teacher would lead the piece by playing it. Students followed the teacher for substantial note, fingering, and rhythm guidance and attention was strongly directed at the teacher not a score. In comparison, non-Suzuki students had a far better grasp of note names and all non-Suzuki students could read music sufficiently to read pieces requested of them.

### 2.3. Lesson Structure

All students were assigned a lesson for each feedback style during one their four lessons. Lessons were randomly ordered within the constraint that they were equally distributed. Lessons were designed to maintain a balance between tasks to keep students interested and learning while ensuring sufficient repetition of tasks to enable effective comparisons. Each lesson, consistent across feedback styles, consisted of the consecutive three parts in Table 1.

**Table 1:** Structure of study lessons split into sections with task, repertoire, key, and time spent within a lesson section.

**Task**	**Repertoire**	**Key**	**Target length (min)**
Scales and arpeggios	G Major or A Major (2 octave)	G Major or A Major	5
Common repertoire	Long Long Ago (T.H. Bayly) or Minuet III (J.S. Bach, Suzuki Book 1)	A Major or G Major (A section only)	10
Unstructured time	Student determined	—	10

*In this table, key refers only to musical segments included in overall intonation results for that part of the lesson section*.

Two of the three included sections consisted of fixed repertoire in order to be able to compare results across lessons and participants more directly. Scales in participants' lessons alternated between G Major and A Major. The common repertoire was always either Bayly's Long Long Ago, a piece shared between Suzuki Book One and the British ABRSM 2016 grade one exam, or Bach's Minuet III. All but three participants were familiar with one or both pieces prior to the study. Though students played the full piece, collated results of a lesson section used only the music's A section so that results for a section are always limited to a single key. Tasks in unstructured time were decided based on what the student needed help with, which may or may not pertain directly to the study.

The amount of time and number of repetitions within each section depended on the student and the lesson length. Students unfamiliar with the chosen common repertoire piece might spend most of the lesson working on it and largely skip unstructured time, while more competent students might speed through required tasks and spend most of the lesson in unstructured time. Similarly, if time was short, sections were moved through faster.

On the whole, 32% of lesson time was spent on scales (18%) and arpeggios (14%), 22% on common repertoire, 38% on unstructured time, and 8% on tasks outside of the study. In a typical lesson, students would play three scales, followed by three arpeggios. As arpeggios were less familiar, these were often repeated additional times. The common repertoire piece was frequently repeated unless a student displayed a high level of proficiency playing it.

#### 2.3.1. Choice of Scale

Within this study we used equal-temperament tuning for detecting error and tuning aural feedback. Expert musicians who do not play instruments with fixed intonation are well-known to use different tuning systems such as Pythagorean, 6th comma meantone, and expressive intonation (Loosen, 1993; Kopiez, 2003; Leukel and Stoffer, 2004; Duffin, 2008; Johnson, 2017). In many of these tuning systems, tuning becomes contextual; the tuning of a note may depend on the key or its melodic role. In contrast, equal-temperament tunings are consistent across all scenarios.

We do not expect beginning violinists to be familiar with the subtleties of different temperament systems, and given the ubiquity of equal temperament on the piano and in wider musical culture (Duffin, 2008, 19), we felt the use of equal-temperament was a reasonable compromise for defining what was correct within the study even if some deviation from equal temperament which would be marked as an error in this study might be positively perceived by an expert listener.

Based on pre-study trials, when the performance task featured pre-dominantly single-key music (scales, arpeggios, and common repertoire), we corrected pitches to the nearest equal-temperament note in the relevant key. It is common for beginners to play an incorrect accidental, and *snapping*, pulling a pitch to the nearest target note, to key meant that the pitch corrected audio should correct to the proper note. As one beginner in stated during pre-trials in response to snapping chromatically:

“I was getting it wrong. I didn't know if I was sharp or flat because it was correcting me to the wrong note. And as a beginner, …I knew it was wrong, but I didn't know why.”

After snapping to key, our beginner reported:

“That was much clearer…I knew I could trust my ear and it was straight to adjusting whether I was flat or sharp. ”

### 2.4. Quantitive Data Collection and Analysis

We logged two timestamped data streams from the augmented violin: one for sensor readings and one for pitch estimation. Sensor readings of finger contact location were logged every 11.6 ms with pitch estimates (as played, as corrected, and as displayed) and audio volume logged every 2.9 ms. Direct audio of the performance and aural guide along with video were recorded for every lesson.

Results for pitch were calculated based on the logged equal-temperament linearized estimates of pitch performed^8^. When calculating intonation accuracy, we removed any pitch estimates logged during periods of low volume, presumably rests of musical pauses, or that were clearly incorrect (namely those above or below the possible played range). We then calculated mean absolute intonation error (MAIE), the mean intonation error, and the root mean square pitch error (RMSE) for each lesson segment. As much of what we are interested in is the intonation correction process, we calculated continuous frame-by-frame intonation error for the duration of a note.

Intonation error was determined as the estimated difference in cents between the pitch played and the nearest chromatic pitch. For example, if A440 is defined as our zero reference point, each equal-tempered semitone will have an integer pitch value, such that the C above (523 Hz) has a value of 3. Thus, a linearized pitch estimate of 3.22 would be considered an error of 22 cents sharp (the non-linearized pitch estimate of 3.22 is 530 Hz).

#### 2.4.1. In Study Algorithm Variation

There was one significant issue with the augmented violin implementation that arose and was addressed during the study. Despite rigorous pre-testing with adults, as lessons with the half-sized violin progressed, it became apparent that it was challenging for non-adults to sufficiently press down the string to trigger the fingerboard sensor when playing the first finger. Due to how our low-latency pitch detection algorithm was optimized, when the first finger was not pressed down adequately, it resulted in a pitch estimate up to 70 cents flat. Once detected, the issue was fixed, but it persisted for exactly half of the lessons. The impact varied by participant and by lesson. Numerical results in the study are based on the revised algorithm but participant experiences may have been affected by which algorithm was used during their lessons.

### 2.5. Qualitative Data Collection: Soliciting Student Feedback

Participants were asked for their reaction to feedback methods by the author/teacher through two sets of semi-structured interviews. Questions were intended to assess whether students benefitted from and enjoyed feedback and to facilitate conversation. Feedback was also discussed within the lesson as teaching situations arose. Planned questions asked after the completion of each lesson were:

**List 1:** 

1. What does it feel like to use the feedback tool?	2. How did you use the feedback?	3. Did anything surprise you?	4. Did you find it helpful?

The last lesson also included a final interview on the overall experience. Feiring conducted final interviews with his students (P1, P4, P7) while at the Suzuki Hub, for practical reasons final interviews were primarily asked by the author. We recognize that having the author/teacher leading discussion with the student is at particular risk of acquiescence bias so when practical, or if a student was being particularly shy or appeared suggestable, additional interviews were requested. Suzuki Hub director Kate Conway re-interviewed three participants and one student was re-interviewed by her mother.

The four overall study questions are listed in List 2. The phrase “one headphone” was used as a colloquial term for aural feedback.

**List 2:** 

1. Of the four lesson types, which was your favorite? Why? [Reminder of lesson types: (1) visual feedback with the colored bar and note name, (2) one headphone with a guide pitch, (3) using both, or (4) no technology, just the teacher!]	2. What was your favorite part of using the augmented violin and why?	3. What did you like the least about using the augmented violin and why?	4. If you were practicing, do you think the visual or headphone feedback would be helpful? When and why?

Lessons and interviews were recorded on video and annotated with transcribed dialog and information about how long each section in Table 1 lasted, whether the student was clearly looking at or away from any visual feedback, and whether the teacher was playing with the student.

#### 2.5.1. Thematic Analysis of Qualitative Feedback

Qualitative results were derived using thematic analysis (Braun and Clarke, 2006). Having extracted information on which feedback method was a student's favorite (in the context of their lesson experiences) and which they thought they might prefer during independent practice, all annotations of conversations and events were reviewed for common themes by the primary author. We conducted two levels of review for themes; the first was themes within or specific to a given feedback method, and the second was themes shared across feedback methods. Comments clearly about a specific single feedback style, such as aural only, or visual only, made during a lesson using combined feedback were considered relevant to the particular feedback method, rather than only in the context of mixed modalities.

Once potential themes were collected, they were reviewed for clarity and significance. Clarity was required not just whether the particular theme was well-defined, but whether the meaning of the intention of the student's comments contributing to the theme were sufficiently clear and independently generated. If a question was deemed too leading or pressured, the student's response was thrown out. Significance was given to themes either repeated by a large number of students, or generated by a strong response by a small number of students. Themes directly from participant feedback form the structure of section 3.2.

## 3. Results

Fourty-nine lessons were completed with 47 lessons included in overall results. Two lessons were repeated due to issues with study execution (one is discussed in section 4.3).

### 3.1. Quantitative Results

Numerical analysis of intonation accuracy was segmented per lesson section into scales and arpeggios, an introductory common repertoire piece, and unstructured time (Table 1). For each participant, we derived the mean absolute intonation error per section with repeated scales, arpeggios, or pieces grouped together within each section. The overall error for a participant's lesson was taken as the mean across all sections. The mean errors from each participant were then used to calculate the mean absolute intonation error across all participants for all sections, as shown in Table 2. Scales and common repertoire are measured against the key of the section and have a maximum error of 100 cents, while error during the unstructured time is measured against a chromatic scale with a maximum of 50 cents.

**Table 2:** Results of study with beginners using augmented violin with variable feedback.

		**Feedback method**			
		**Aural**	**Aural & Visual**	**Visual**	**None**
Overall	MAIE	19.81	18.59	18.12	**17.93**
	STD	(4.79)	(3.32)	(4.13)	(4.32)
	Lowest Err	2	3	4	3
Scales &	MAIE	22.96	22.20	20.37	**19.97**
Arpeggios	STD	(5.84)	(4.97)	(6.74)	(6.07)
	Lowest Err	3	3	3	3
Common	MAIE	17.75	**16.56**	17.63	16.77
Rep.	STD	(6.25)	(4.89)	(6.76)	(5.93)
	Lowest Err	2	4	4	2
Unstructured	MAIE	18.01	**17.03**	17.06	17.64
Time	STD	(3.37)	(2.88)	(2.88)	(2.38)
	Lowest Err	2	3	4	3
Preferred lesson type		**7**	2	2	1
Preferred practice type		0	**8**	2	2

*MAIE is the mean absolute intonation error with STD the standard deviation between participants. Both MAIE and STD are in cents. Error was measured against key except during Unstructured Time which was measured chromatically. Lowest Err is the number of participants for whom the given feedback method resulted in their lowest MAIE for that section. Numbers for preferred lesson or practice represents the number of participants selecting the given feedback method as their favorite. Lowest MAIE per section is in bold*.

Our experimental data tracking performed pitch did not provide evidence to support our hypothesis that overall, a beginner violin student will perform with better intonation provided with aural, visual, or both forms of feedback. We used a two-way repeated measures ANOVA to check for statistical significance (*p* < 0.05) for differences between feedback types and study section. Though the difference in performance during study section did meet significance criteria [*F*_(2)_ = 17.73, *p*≪0.01], we did not find any statistical difference between different feedback types [*F*_(3)_ = 0.83, *p* = 0.48]. Considering the overall mean absolute intonation error for each of the four feedback cases, the maximum difference between cases was only 1.88 cents (between no feedback and aural feedback, a 10.49% difference), considerably smaller than the standard deviation within any of the four cases. With such high variance, we would need a much larger sample size for conclusively determining whether our hypothesis was true or false.

**Figure 3:**
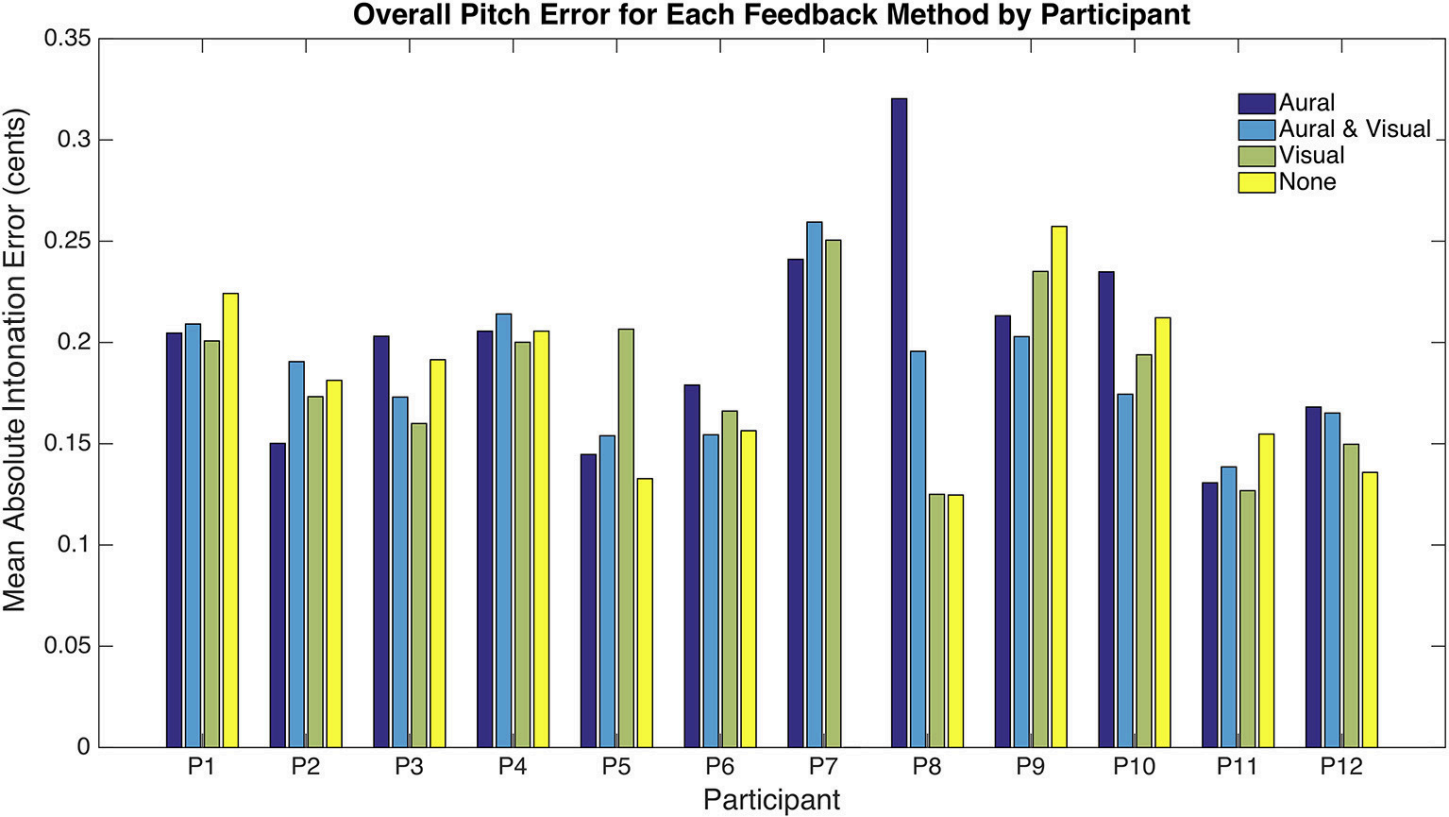
Overall mean absolute intonation error per lesson for different feedback methods for each participant.

Figure 3 depicts performance results on an individual basis, highlighting the divergent response of participants to different feedback mechanisms. The overall means for each feedback condition masked a significant variation in performance both between participants and within an individual's set of lessons (Figure 3). More meaningful than statistical analysis in our case is to look at trends amongst participants. For instance, P8, performed dramatically worse in the lessons with audio feedback than without. In fact, P8 accounted for a substantial amount of the overall difference in performance between aural feedback and no feedback cases in Table 2. Removing P8, the difference drops to 0.35 cents (1.89%). Though no certain conclusions can be drawn, it is reasonable to hypothesize that for P8, aural feedback had a negative impact on intonation performance.

#### 3.1.1. Student Preference

As in section 2.5, upon completing the study, students were asked both what was their favorite lesson, and what feedback they would find helpful during practice (if any) (List 2). Individual responses are given in Table 3.

**Table 3:** Participant stated preferences for favorite lesson type and what feedback method(s) they speculated they would most like during individual practice.

	**Participant**											
	**P1**	**P2**	**P3**	**P4**	**P5**	**P6**	**P7**	**P8**	**P9**	**P10**	**P11**	**P12**
Aural		L	L	L	L	L	L			L		
Aural & Visual		P	P	P	P	P		P	L		P	L, P
Visual	P							L	P		L	
None	L						P			P		

*L means participant responded the given feedback method was their favorite lesson style, while P means given feedback method was their preferred option for potential use during practice*.

**Figure 4:**
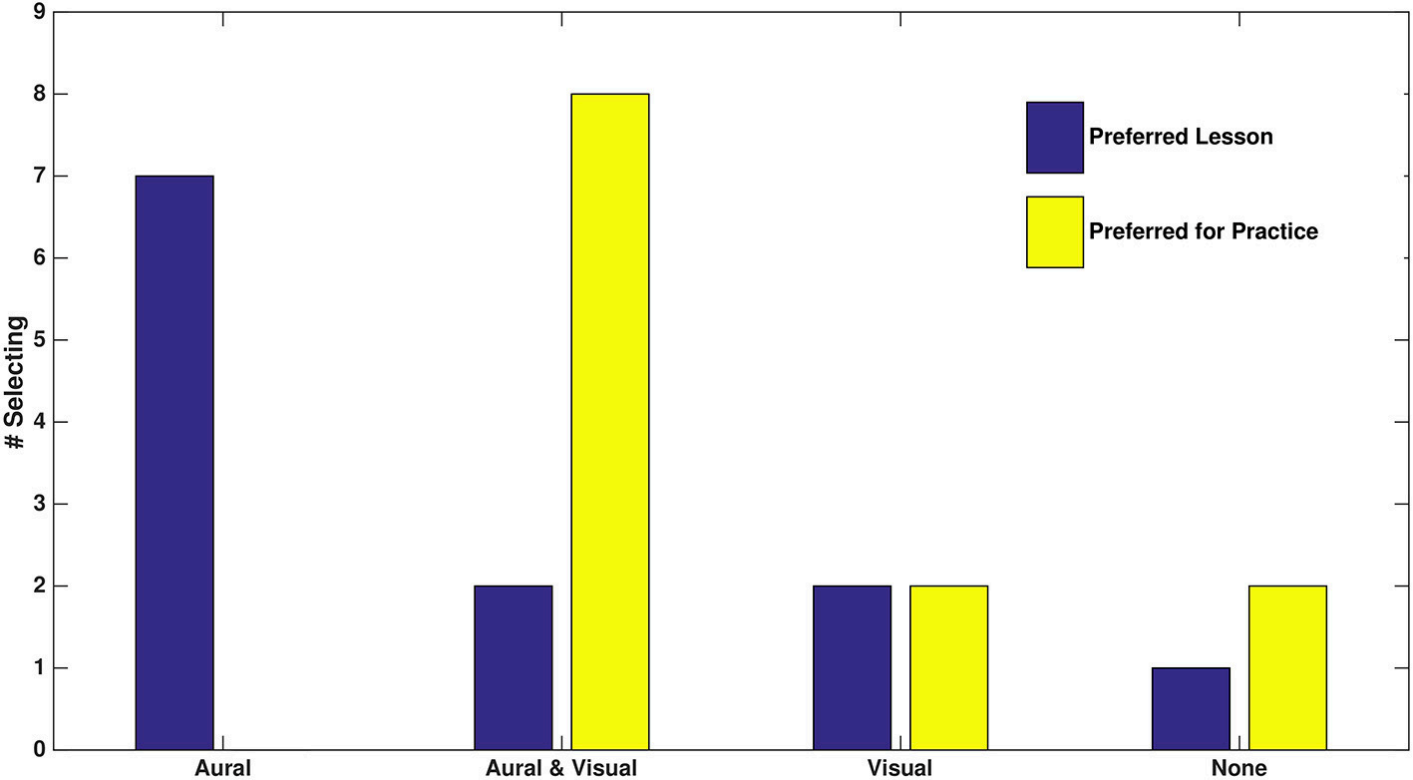
Reported preferences of the 12 participants for favorite lesson within feedback types and what feedback option(s) would be most helpful during practice.

Fifty-eight percent of participants reported aural feedback as their favorite lesson type (Figure 4). Only one student reported preferring no feedback with two opting for visual and two for combined feedback. While the question about favorite lesson type seemed to lead to single modality answers, when changing the question to what a student thought would be most helpful during practice, two-thirds of participants stated a preference for having aural and visual feedback. Two participants reported they would probably only use visual feedback during practice, with one (P1) explicitly saying he did not like the aural feedback as, though sometimes he found it useful, it was also distracting and he did not trust it. Two participants reported they were not interested in having any intonation feedback method while practicing.

With such a small sample size, performing *p* < 0.05 significance testing risks a high likelihood of a false negative, meaning that while we can not make any assessments with statistical certainty, it is again valuable to consider trends within responses. Combining responses from both questions about feedback preference in Figure 4, aural feedback was included in 70.8% (17 of 24) of participant's choices, with visual feedback included in 50.0% (12 of 24) of responses. With no feedback only chosen in 12.5% (3 of 24) of responses, trends suggest that beginners do indeed find value in both aural and visual feedback.

### 3.2. Qualitative Results: Thematic Analysis of Student Responses

While quantitatively it is difficult to make any definitive conclusions whether aural and/or visual feedback are effective for improving a student's intonation accuracy, we obtained a wealth of qualitative reaction through annotated casual discussion, interviews at the end of lessons (List 1) and upon completing the study (List 2). We split review of participant feedback and responses into themes. After a brief discussion of bias and we look at some of the primary themes that arose: utility, attention, familiarity, sensory overload, immediacy, and trust and authority.

#### 3.2.1. Potential for Bias

Prior to discussing user feedback, as suggested in section 2.5.1, it is necessary to point out that qualitative responses may be susceptible to bias, and unclear or unreliable statements. For instance, answers may favor more recent lesson types or be influenced by interviewer comments. There is evidence of this within the study such as numerous contradictory statements, typically between different lessons. For instance three participants stated at different times they liked either aural or visual feedback better than the other only to reverse their preference^9^.

With those caveats, we assume that students are truthfully reporting their experiences. In interviews, we always endeavored to ask students to justify and explain simple answers, not only in an effort to gain wider insight, but also to make sure they could provide reasonable rationale for their answers. Without rationale, we did not weigh yes or no answers strongly. Despite efforts to encourage forthright responses we still expected a level of acquiescence bias as students generally sought approval from the author/teacher. Alternatively, we found examples of students (P1, P8) taking a contrarian stance, only reluctantly admitting to use of feedback.

#### 3.2.2. Comments on Effective Utility

While aural feedback resulted in the worst intonation accuracy for 5 out of 12 participants (P3, P6, P8, P10, P12), it was by far the most popular lesson type (7 out of 12). In response to questions from List 2 and question derived from List 1, five participants (P2, P3, P5, P11, P12), asserted that, as intended, aural feedback highlighted error and provided practical feedback for correcting. For instance, P4 stated, “you realize how much you go out of tune and so it helped me to stay in tune.” Another, in response to whether the aural feedback was useful, said:

“Especially with the shifting. Because it sort of tells me the right note, like the equivalent to what I would be playing. Except I'm doing it for a shift so if my shift is out of tune it tells me the note. Which helps a lot.” (P5)

Similarly, asked the same question, P11 responded, “Yes, because I wouldn't know where to go if I didn't have that. If I notice that I'm playing out of tune, it tells me whether I'm flat or sharp.” Having already done both the lessons including aural feedback, during the no-feedback lesson P11 remarked, “There was a bit where I didn't know if I was flat or sharp and then I missed [the aural feedback] …I admit I missed then having the guide.”

Participants also generally commented positively about visual feedback (10 out of 12) as it also informed participants whether they were in tune. The graphic design was praised as easy to interpret: the colored block below the in-tune line meant raise the note, and above the line meant lower it. P5 remarked, “…I quite like the fact that you can look at it and see, oh, I'm a bit sharp, or a bit flat. Even if it is just slightly and you can't hear it and it is good for getting it really in tune to perfect a piece.”

Participants overwhelmingly selected aural and visual feedback as their hypothetical preference for practice (8 of 12). Seven participants valued the ability to switch between feedback options depending on needs (P2, P4, P5, P8, P10, P11, P12). When aural feedback was confusing, having visual feedback allowed the participant to clarify what was happening, and having aural feedback was useful when playing with a score or unable to devote sufficient visual attention. P8 stated, “[visual feedback] is helpful as well because when it's red you know the options…if you take away the headphones I would see, if you take away the visuals I would hear from the headphones.” Visual feedback was reported to add clarity to aural feedback on how to correct as well as external validation about how the participant was performing (P11, P12). As P11 said,

“It's nice to have the visual on, but in terms of actual playing I would use the headphone and my ear and then every now and then I'd look over and see if I was getting a green on the *C♯*.”

Two participants, expressed a preference for no feedback at all in both the lesson and hypothetically during practice with P1 explaining, “I liked the person because the person can tell you exactly what you did wrong but computer and headphones they can't tell you.”

#### 3.2.3. Attention

A major problem with visual feedback was that it required active visual attention. In order to use visual feedback, the student must be able to look at it. Half the participants (P1, P2, P4, P5, P11, P12) confirmed that it could be challenging to use visual feedback when visual attention was required elsewhere. When asked why he was not using the graphics, P1 responded, “Because if you look at it, you can't see what notes you're going to play also at the same time.” P2 confirmed further saying, “Let's say if I'm learning a new piece I probably won't be able to look at [the visuals to see] if I'm on the right note because I'm [looking at the score] and I'm obviously checking my fingers, and I've already got 2 or 3 things to look at.”

Gaze annotated during lessons, a sampling of user visual attention, suggested during lessons using visual feedback, the graphics did not regularly receive attention. Of annotated periods in lessons where visual feedback was available, participants were clearly looking in the direction of visuals only 16% of the time. In comparison, 59% of the time participants appeared to be looking at their violin, and 19% of the time they were looking at a score. Though indicative of use, our simplified measure of gaze is not necessarily fully inclusive; for instance, P4 remarked he watched the feedback out of the corner of his eye while focusing on the score. Participants most commonly looked at visual feedback during scales and arpeggios which were taught aurally and did not require use of a score.

As a result of issues with visual attention, the consensus amongst the two adult participants, P2 and P11, was that visual feedback was only an effective modality of feedback once the notes of a piece had been learned. When starting a new piece, wearing the headphones was far more useful.

In contrast, four students expressed that they appreciated that aural feedback was essentially a passive yet present form of feedback (P2, P4, P5, P11): using it did not require an intentional direction of attention. P5 remarked, “you sort of don't really know that it's actually playing, because well when you're in tune it sort of melds into your playing.” However when acoustic and corrected pitches clash, it stands out. As P11 said, “[Aural feedback is] really helping, it sounds horrible when it's out of tune, but when I get it's like, ah yeah!”

Still, part of making aural feedback effective and ensuring attention is appropriate volume. Too soft and the participant can not use it effectively, yet too loud and it becomes painful and distracting. For instance, P2 pointed out in a lesson that he could not hear the guide sufficiently and requested it to be louder, while P8 had the volume distressingly loud for most of lesson.

#### 3.2.4. Sensory Overload and Rejecting Feedback

Sensory overload was an issue for both aural and visual feedback. Auditory overload was pointed out by P11 who stated that when the author/teacher played with her, she had too many conflicting versions of audio to effectively respond to. When asked what was her least favorite part of using the augmented violin, P11 replied, “I remember when I had the headphone, and me, and you were playing and that was way too much, and you were triggering wrong notes and stuff.^10^”

Alerted to the issue, the author/teacher responded by playing less in lessons, however the author/teacher still played with students 47% of the time. In cases where the student expected the teacher to play with them, primarily in lessons with Suzuki students, the author tried to play quietly so as not to interfere with or overshadow the aural feedback, however it may have still been problematic.

Additionally, while aural feedback audio was generally of good quality and free from artifacts, it still sounded different. Two thirds of participants commented that the feedback audio sounded funny (P1, P3, P4, P5, P6, P8, P10, P12) or that wearing headphones felt odd. Besides tonal differences, not only were there times when it burbled on low strings as a result of pitch tracking error, but ambient sounds from the room were amplified and potentially pitch corrected as well. Students commented “Everything sounds weird now (P8)” or “Everybody sounds like a robot (P3)”.

Participants were told they were free to remove headphones if they wanted. Two students requested to remove the headphones. P4, who liked the aural feedback but would frequently remove his headphones when not playing, explained there was background noise which he found unpleasant and hard on the ears. In future, efforts need to be made to eliminate or reduce background noise in aural feedback.

Only P8, requested to take them off fully: once as it had been too loud and the other time he stated he disliked the aural guide, the headphones were bulky and the cable annoying. In both cases he completed all but the unstructured time wearing headphones. P8 was also the one participant whose intonation accuracy was dramatically worse in lessons using aural feedback (see Figure 3).

Similarly, visual feedback also suffered from sensory overload. Half the students expressed that visuals were distracting due to feedback changing too rapidly for users to process and flicker due to estimation error between notes. For instance P11 remarked,

“[The visual] is useful. It's interesting. Obviously the color and the direction of the bar does help, but in a playing situation, it's way too much and it's flickery and it makes me just really confused, whereas if i just focus on what I'm hearing it's much easier to play it in tune…it was all red bars everywhere and it was going too fast to correct it whereas if I'd of been able to hear it I could have corrected it.”

Another issue with visual feedback, noticed by P2, is that his reaction time to visual feedback was slower. Above some tempos, aural feedback will also be unusable, but aural feedback has the advantage that we can process auditory stimuli faster. It takes 40μ*s* for the brain to convert sound to neurological signal (Hanson et al., 2009). In comparison, it takes 50 ms for humans to trandsuct visual stimuli in normal light. Hanson et al. (2009) studied reaction times to different modes of interaction finding that people react noticeably faster to auditory stimuli than visual stimuli (161.3 vs. 206.9 ms in unimodal trials).

Technologically, audio delay was also less than graphics. The audio latency of the pitch-correction software was 11.6 ms in comparison to the screen update which was roughly every 100 ms. Further, intonation data required a low-pass filter to be stable enough for human perception.

Combined feedback will suffer from the same sensory overload problems that both aural and visual feedbacks have independently. The combination will not fix flicker in visual feedback, audio noise in the aural feedback or potential overstimulation, but having both does give the participant the option to switch focus if desired. In fact, there was some evidence that trying to use both feedback methods simultaneously was itself overwhelming (P2, P11).

#### 3.2.5. Familiarity

Familiarity turned out to play a significant issue for both feedback methods, but in different ways. Despite largely positive response to aural feedback, both adult participants mentioned it was necessary to habituate oneself to having an additional audio source and manage potential sensory overload. P2 highlighted, “I think it's because I'm trying to listen in a different way…” and P11 stated, “I need longer to get used to this way of playing and I think it'd get easier.”

Both adults expressed the desire to play more with the aural feedback to “get used to it.” Learning to balance focus between the acoustic violin and the headphone feedback was a distinct change from unaugmented practices.

Strong evidence that aural feedback takes some acclimation comes from separating lessons further into component parts, splitting intonation performance between scales and arpeggios (Table 4). As stated in section 3.1, we found a statistically significant difference in performance between sections and though we can not separate how much of this difference is due to task, warming-up, or novelty of feedback, data suggested the introduction of aural feedback may also have had an initially disruptive effect.

**Table 4:** Reduced version of Table 2 giving the MAIE of different sections with scales and arpeggios separated.

	**Lesson primary feedback method**			
	**Aural**	**Aural & Visual**	**Visual**	**None**
Scales	23.24	22.81	20.12	**20.05**
Arpeggios	22.01	20.74	20.03	**19.54**
Common Rep.	17.75	**16.56**	17.63	16.77
Unstructured Time	18.01	**17.03**	17.06	17.64

*Improvement in intonation error throughout the lessons without aural compared to reduced improvement in cases with aural feedback suggests it may take time for a user to become accustomed to aural feedback. Lowest MAIE per section is in bold*.

In both methods using aural feedback, intonation accuracy during scales was more than 2.5 cents worse than other methods. This gap is the largest difference between group means seen in this entire study. Intonation in aural inclusive methods improved in comparison to non-aural methods during arpeggios, and by the common repertoire section, intonation in aural inclusive modalities improved to the extent that they were largely on par with non-aural modalities. Improvement between scales and common repertoire for aural feedback inclusive methods was nearly double the improvement seen in both non-aural cases.

Some of this difference may be that, while visual feedback uses a sensory modality which is not strictly required for performance, aural feedback alters a key, already in use mode of feedback. Participants must become accustomed to hearing their violin in only one ear while hearing a compressed pitch corrected version which does not necessarily match their playing in the other. While participants were used to playing with teachers and hearing two people playing, participants reported aural feedback was quite different (P8, P11).

“I preferred the feedback to your violin: audio and visual. It was much easier to hear and see. Because of the compression, I could tell which one I was aiming for really clearly. When I had got into it, I was getting to grips with the feedback and was getting much better at correcting my pitch.” [P11]

Familiarity was a challenge with visual feedback for a different reason: unfamiliarity with note names. Participants found the basic bar graphic described and pictured in Figure 2 easy to use, but note names were of limited use. Only 3 study participants, (P4, P6, and P11) showed competence naming notes in a score. All other students referred to notes by their first position fingering. Even more experienced players displayed poor knowledge of note names. This is an area we expected Suzuki students to be weaker and indeed, despite making up two-thirds of participants, only one of the students recognizing note names was a Suzuki student.

The problem with note unfamiliarity is that most students could therefore not determine whether they were playing the correct note or not. Unlike aural feedback, visual feedback did not follow a diatonic scale as participants were expected to recognize the difference between a *C♯* and *C♮*. This expectation usually proved untrue thus reducing the usefulness for correcting highly out of tune notes.

However two players pointed out displaying previously unfamiliar note names may provide a learning opportunity. P8 stated “I would [use the visual feedback] so I know F# and A#. I will know the notes; how to say them in the letters.”

#### 3.2.6. Explicit vs. Implicit

Participant comments suggested that one reason for liking visual feedback over aural feedback was that it was more explicit. Three users (P2, P8, P11) expressed that it was sometimes difficult to decide which way to fix a note using aural feedback whereas with visual feedback it was easy to interpret as it showed in which direction to move their finger. P11 stated:

“Think I liked [visuals] the most. It's enough information for me to correct it…like I knew I was out of tune when you were just teaching me without anything, without any aids, but I didn't know how to correct it, whereas this is really clear how to correct it…[With] the headphones, I could hear that I was out of tune and that made it quite stressful, where as [with visuals], there is only one thing I'm listening to, it is just my note which is easier to process and really clear …whether I am too high, too low.”

P5 also suggested that aural feedback could be stressful as one might hear they were wrong but did not always know the best way to correct it.

#### 3.2.7. Trust and Authority

Trust is crucial for intonation feedback tools to be useful. If a practice aid is frequently wrong, it loses its value. Students appeared to generally trust the intonation feedback with P4 stating a reason he liked the feedback methods was because, “They give you true feedback, they don't always give you positive.” As the author was an expert and the beginners were working with technology, there was a high degree of inherent trust that the technology the author/teacher was offering was trustworthy. This was true even as it struggled to correctly identify the pitch of the three lowest notes on the violin. Asking P6, who has perfect pitch, about visual feedback at the bottom of his scale:

A: “Do you think it is right at the end?”

P6: “I think it has to be.”

A: “Actually no, at the bottom it gets it wrong and you get it right.”

Trust and authority were necessary to encourage participants to use feedback, but they had the drawback that participants were less likely to report issues during the experiment. For instance, with aural feedback, there were multiple times the author initially failed to correctly set the key for aural feedback, yet only two participants (P4, P11) ever pointed the mistake out. Similarly, if headphone volume was uncomfortably loud, rather than speaking up the participant would often tolerate it through out most of the lesson or until the author sensed something was amiss (P8, P9).

Despite trust being high, two participants (P1, P8) expressed they thought the system was sometimes wrong. Neither of them said they liked the aural feedback, with P1 preferring no feedback at all.

### 3.3. Observational Case Study: Referencing of Aural Feedback

Active use of aural feedback is difficult to assess outside of self-reporting since listening is not externally visible. As an observer, it can be difficult to infer whether an improvement in intonation accuracy is due to the enhanced feedback tools, the student's self-assessment based on their internal memory of the task, teacher instruction, or another contextual factor. Especially as out quantitative data is inconclusive as to whether aural feedback has any effect on intonation error and therefore any indication whether it is being used or not, it is valuable to confirm its active use outside of self-reporting.

Being a manual intervation, there were occasional instances in the study where the key for a task was temporarily set incorrectly. Repeated or uncharacteristic intonation error triggered the primary researcher to ask whether the key was set correctly. Though sometimes correct, sometimes it was not. In most instances, the error in intonation was neither severe nor sustained, so while suggestive of active use of aural feedback, it was not strongly indicative of use. However there was one instance where a mistake setting the key correctly led to strong evidence that a participant was actively following and using aural feedback.

**Figure 5:**
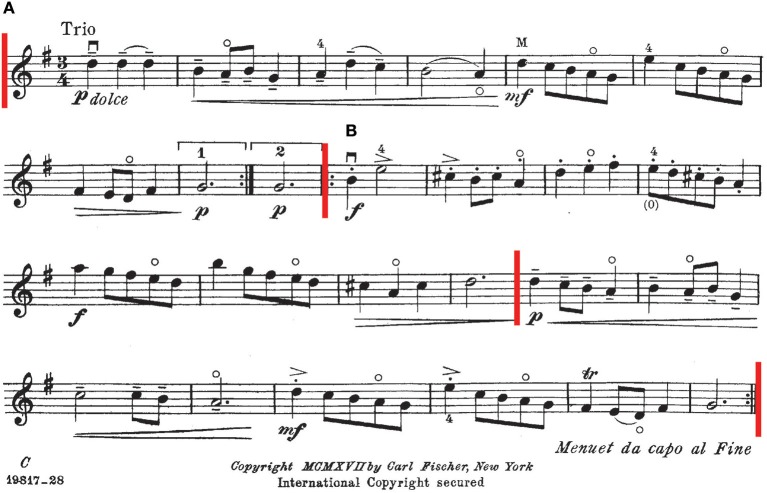
Score to Bach's Minuet 1 (arr. Seely-Brown). The piece begins with an A section in G Major. The second section, B, is composed of a section in D Major followed by one in G Major. A red bar marks the change in key along with starts and ends of sections.

P9 was shy and rarely provided any exposition about her experiences within the study but was perceived as a diligent student. During her last lesson, which was using aural and visual feedback, she worked on a Minuet by Bach in G Major, shown in Figure 5 and which she was playing by memory. Previously we had been working on a task in A Major, and the researcher forgot to change the key for aural feedback to the new key. As the aural feedback was still in A Major, the aural guide incorrectly snapped all *C♮*s to *C♯* and *G♮*s to *G♯*.

**Figure 6:**
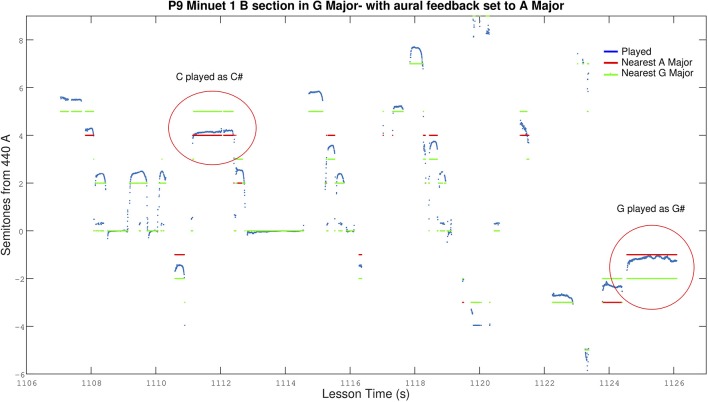
Section of P9 playing Bach's Minuet 1. Aural feedback is incorrectly set to A Major and P9 can be seen playing closer to *C♯* and *G♯* which feature in A Major, but not in G Major. Red lines represent the nearest note in A Major, and green lines the nearest note in G Major. In the case of the circled *C♯*, the note should be *C♮* but as the played note is sharper than *C♯*, the nearest note in G Major is D.

P9 proceeded to play the Minuet on her own with a mixture of *C♮*s, *C♯*s, and *G♯*s. As she continued playing, intonation error steadily increased, until mean error had risen from 27 cents initially, to 40 cents. Figure 6 illustrates a segment of her playing while aural feedback was incorrectly set to A Major. The expected *C♮*s (3 steps above A) were closer to *C♯*s (4 steps above A) and the intended *G♮* (2 steps below A) was performed much closer to a *G♯* (1 step below A).

As P9 had played the Minuet in previous lessons with the correct accidentals, the uncharacteristic error caused the researcher to check the key and notice the incorrect *C*. After restarting the piece, this time with the researcher playing along to reemphasize the correct accidentals, P9 started fixing the *C♮*s, but continued to incorrectly play *G♯*s.

Not realizing the aural feedback was still was giving a *G♯* as the guide note, the researcher as teacher, stopped her playing, pointed out the incorrect accidental, played her the music correctly, and asked her to play again. This process repeated a few times as P9 continued to play with a *G♯* and the researcher continued to interrupt and correct her. Baffled why an otherwise attentive student who normally tried to follow instructions was not making a correction that was seemed within her skill level, the reseracher double checked the key setting and realized the mistake. The aural guide was still was snapping to *G♯* instead of *G♮*.

**Figure 7:**
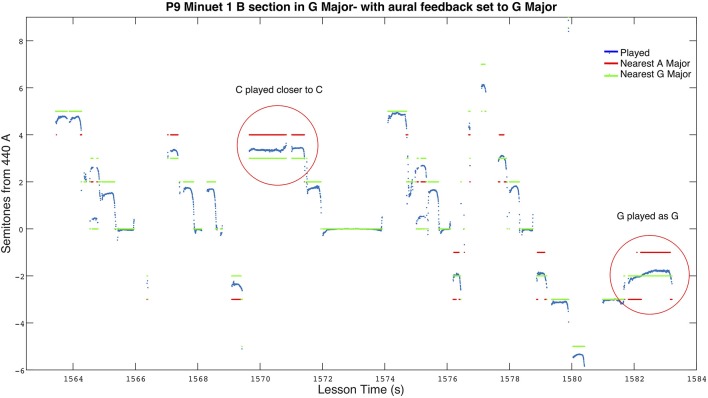
Section of P9 playing Bach's Minuet 1. Aural feedback is correctly set to G Major and P9 can be seen using *C♮* and *G♮* instead of *C♯* and *G♯*. Red lines represent the nearest note in A Major, with green lines the nearest note in G Major.

Upon finally fixing the aural feedback to the fully correct scale and asking P9 to play the problematic parts again, P9 perceptually began playing closer to *G♮*. Figure 7 depicts P9 playing the same section shown in Figure 6, but with the correct aural guide. The fixed *C♮*s and *G♮*s resulted in an improved intonation accuracy of 22 cents mean absolute error, an 18 cent improvement over when she was playing with the incorrect guide.

Through the accident of incorrectly set aural feedback, we can see strong evidence suggesting P9 was following aural feedback. In spite of direct repeated instructor intervention, and prior aural memory of the piece, P9's *C*s and *G*s perceptually followed the audio feedback guide. While the error in the aural feedback setting would have increased her intonation error when she was using it, if she was successfully following a correctly set guide at other times, we would expect her intonation accuracy to be better in lessons with aural feedback. Though not statistically conclusive, P9's broader results in Figure 3 support the idea that P9's intonation benefited from aural feedback as both lessons including aural feedback resulted in the lowest intonation error for P9's lessons.

### 3.4. Reflections From Teacher's Perspective

As the teacher conducting lessons and watching the use and reactions of students to the different feedback methods, my impressions, though biased by knowledge of the research objectives, remain relevant and potentially insightful. Overall, students were very positive toward all methods of feedback. Just as Johnson found in her studies of violin practice aids (Johnson et al., 2012, 2013), feedback preferences varied between students.

#### 3.4.1. Perceived Reaction and Use of Feedback Aids

Students liked aural feedback for the reasons we expected: it helpfully highlighted error, giving the correct version that most students could intuitively follow once they got used to it. It was hard to tell how much students were using it, leaving me sometimes surprised that some students did not correct themselves. It is also appeared that once the algorithm for pitch detection had been altered to capture first fingers more easily (section 2.4.1), reaction to aural feedback became more positive.

Although it is not evident in the numerical data or student quotes, from my teacher's perspective it appeared that very few students genuinely used the visual feedback. Even though we annotated gaze suggesting graphics were used 16% of the time, I did not get the impression many students were responding to it. Both adults, P2 and especially P11, seemed to make a continuous effort to look at and respond to the visual feedback, but otherwise, despite comments saying they liked it, only three children (P3, P6 and P8) visibly directed attention beyond the very start of the lesson when I presented how to use it. Apart from P11, and possibly P4 who said he kept the visual feedback in his periphery, my impression was no one (including P2) used visual feedback beyond the first section of the lesson, scales and arpeggios.

As a result, my subjective impression was that lessons with visual feedback were effectively the same as lessons with no method of feedback. That is not to say visual feedback is not potentially a useful tool, only that in the lesson context with me present, I believe only one or two students used visual feedback significantly.

#### 3.4.2. Teaching With Feedback Aids

As a teacher, I found both methods of feedback useful as teaching aids, visual feedback more so than aural. Aural feedback was effective as a concept but I could easily play with the student to achieve similar effect. Visual feedback however was useful in pointing out major error or discussing with the student what note they should be playing, similar to how the teacher used visual feedback in Menzies (2015, p. 125).

Though overall I felt visual feedback was a useful teaching aid, there were times it was more of a distraction. With some students, while giving oral instructions, I was worried about losing their focus to the visual graphic (P6). Another time, a student struggling to play a piece from memory seemed more prone to losing her place when she looked at the visual feedback.

Aural feedback was also prone to causing confusion if I forgot to set the key correctly. I often could not hear the pitch corrected audio so that it was only when students repeatedly played closer to the wrong accidental that I caught my mistake (P1, P8, P9, P10). Though causing rather than reducing error, this did suggest they were responding to the feedback. Still, when set correctly, aural feedback seemed more help than hinderance.

#### 3.4.3. Practice vs. Lessons

One final reflection is that though we tested in a lesson context, these feedback methods were designed with practice, not lessons in mind. Aural feedback was not particularly necessary in the lesson as I could just as easily have provided an aural guide by playing with a student with the added benefit that they could watch my fingers. Students often expected me to play with them which, as P11 pointed out in section 3.2.4, actually undermined the aural feedback. However at home, I am not there to play with them allowing aural feedback to fill the gap.

Additionally, I was regularly challenging students with hard tasks and would judge the result. Pressure to immediately achieve tasks meant students would often do what was expedient and familiar, rather than take the time to focus on the feedback tools. Practice can be much more experimental.

Time was a major constraint and at a premium when conducting the study as lessons. Two 30 min lessons with each feedback method is too short to really test the impacts of intonation feedback. I believe a better judge of our feedback methods would be to build additional instruments and distribute them for a similar length study, but used during practice.

## 4. Discussion

Quantitatively it is difficult to make any definitive conclusions whether aural and/or visual feedback are effective for improving a student's intonation accuracy, however user feedback and our case studies suggest that aural, visual, and combined aural and visual feedback may all be helpful for learning intonation. As previously discussed in section 2.1, “in the wild” studies often lead to more ambiguous results than laboratory studies due in large part to difficulties controlling for external factors (Rogers, 2011; van der Linden et al., 2011a; Kjeldskov and Skov, 2014), and indeed, during our study we saw variations in intonation accuracy which could likely be explained in part by external factors such as student practice outside the lesson, concentration on the day, an unfamiliar violin size (Morreale et al., 2018), and more. More informative than our quantitative results are the comments in section 3.2 and like one from P11, discussing why she liked having extra feedback,

“…it really clarified what I was aiming for and what I needed to do, i.e., move my finger up or down the string. When I didn't know what to do, it was frustrating and overwhelming.”

Fleming and Baume (2006) proposed a popular model that classifies people as visual, aural or kinesthetic learners. Though the model's validity is a subject of continuing debate (Sharp et al., 2008), it is likely that different people will respond more readily to different modes of feedback. For instance, P11, who in her final interview expressed preference for visual feedback over aural feedback, remarked,

“I think. the reason probably I prefer [the visuals] is that the hearing thing stresses me out because my ear isn't used to it…and the reason it stresses me is that, with my instrument, percussion, I don't usually have to think about [listening], whereas maybe this actually would be good training for me, for my ear, it would probably be good to not have the visual and try and actually hear what I'm aiming for.”

Similarly, based on the aural-centric style of teaching in Suzuki, we might expect Suzuki students to respond better to aural feedback than non-Suzuki students. However we failed to see any clear evidence of this within the study.

Considering our goals are real-world tools for learning, despite participant's positive comments and considering the lack of conclusive statistical data, it is clear there are still many relevant questions about efficacy, such as whether responses were due to novelty, whether feedback will yield persistent improvements, as well as further investigations into how we can improve our methods and tools.

### 4.1. Tool or Toy?

An important question to ask, especially as most of our participants were children, is whether positive responses were due to novelty. Due to the time and resource limited nature of the study, we can not claim participant enthusiasm would persist with more exposure. However there were reasons to believe the feedback methods were viewed by participants as a legitimately useful learning tool. In response to study questions, many players responded in ways demonstrating an understanding of why and when a feedback method was useful.

For instance, P8 reported, “I like [the visuals] because I try and make it as green as possible. I don't like it when it's red, because I don't like to make a mistake on the violin. It kinda annoys me. Plus green is my favorite color.” Liking the color green is a trival response, however P8 started by explaining how he would use the visual feedback effectively to minimize mistakes on the violin. Further, P8 expressed interest in using the displayed pitch names depicted to help learn notes (see section 3.2.5). Additionally, which feedback modality a participant liked most did not necessarily correspond to which they thought was most helpful.

### 4.2. Persistence Effects

It is important to point out that in this study, we did not test whether benefits from using either form of feedback were retained once removed. O'Connor (1987), Percival et al. (2007), van der Linden et al. (2011b) all express concern about making decisions based on a correction modality not normally available during performance, with Percival skeptical of using real-time visual feedback when learning musicianship in particular. Pedagogs similarly caution against using visual markers (Kreitman, 1998, p.51), with violinist Martin critiquing (Reel, 2014), “Dots become a visual crutch that students depend on; they're not listening to themselves, they're just going visually.”

Indeed, Dyer et al. (2015) discusses how it is common that studies using augmented feedback to assist motor learning only aid learning while the aid is present and why this may happen. However Dyer notes that aural feedback has proven an exception, with examples of aural feedback providing lasting learning effects. As our aural feedback theoretically aligns well with real-world tuning tasks and strengthens a student's audiation by passively reinforcing what should be correct, something recommended by pedagogs (section 1.1.2), we would expect aural feedback methods to improve intonation in the long term, but can not make any scientific conclusions without a longitudinal study.

### 4.3. Polite vs. Impolite Feedback

Though we attempted to ensure aural feedback sounded smooth and did not glitch, there is evidence that such an approach might not be optimal. Both adults admitted to not listening to the aural feedback at times. P11 commented, “Yes [aural feedback] is useful, because I can hear it being [the correct note]. Because I can hear it right, it almost means I don't correct.” If a student does not correct, the aid becomes counter-productive. However, the study included two lessons where audio glitching when out of tune caused a strong corrective reaction in users.

Due to incorrect setup, there were two lessons [one subsequently repeated with normal settings (P2)] where audio artifacts occurred depending on how out of tune the player was: the more out of tune, the more noticeable the artifact^11^. P2 said, “It's almost irritating, you wanted to do something about it…if I played the wrong note it [was like it] gave me an electric shock.” P2 subsequently repeated the aural feedback lesson with the input levels fixed stating:

“[The normal sound] does help, it's more clear when you play the wrong note without being annoying, because [before] it was like, oohhh, but, I don't know which one is better; probably the annoying part is good because then it forces you to not miss that note.”

It would be interesting to investigate whether impolite highlighting of error would be more effective at helping students improve or whether the unpleasantness would instead lead to students not wanting to use the aid at all.

### 4.4. Future Potential

Two complaints about our feedback systems were related to noise. Our low-latency visual feedback generated complaints that it changed too rapidly turning it into a potential distractor, while the aural feedback suffered from occasional glitching and created substantial noise when a participant was not playing. At the time of writing this article, we have already done substantial work on addressing these problems.

In this study, we used an augmented system using fingerboard system and a microphone for audio input. We have since experimented with augmentations through magnets that passively induce voltage in relation to string movement yielding audio signals that are cleaner and dominated by the fundamental frequency (Buys and McPherson, 2018). This allows extremely accurate low-latency pitch detection without the need for a fingerboard sensor. Both our augmentation techniques used within the study and the magnetic system can be temporarily added to any violin, and the new approach should be largely trivial to mount only when needed. Between the new augmentation approach and upgrades to software, we have eliminated noise in both aural and visual feedback when the system is not being actively used.

While this study focused on beginners, we expect aural feedback to be useful for more experienced violinists too. Intonation is an ongoing challenge throughout a violinist's career and being able to hear correct intonation in real-time while playing freely is likely to be an asset to any violinist. To our knowledge, no existing tools allow this freedom. Existing tools are either tied to score following (and likely to be slow or reflective only), or, like using a tuner, too slow to be practically useful in all but limited cases. One of the highly practical potential uses for advanced players is that the aural guide can be programmed to assist learning alternative tunings, such as just intonation or 6th comma meantone.

## 5. Conclusions

We ran a four lesson *in-situ* study with 12 participants in order to investigate the potential for real-time technology to act as an intonation practice aid for violin. Participants tested four different types of intonation feedback: (1) aural feedback in the form of a headphone over a single ear providing a guide pitch created by pitch correcting the user's playing to the nearest note in the scale, (2) visual feedback in the form of a graphic displaying the name of the note the user is playing and colored bars depicting level of intonation error, (3) combined feedback providing both the aural and visual feedback, and (4) no feedback beyond a traditional violin.

Participants generally responded positively to all types of feedback though statistical analysis does not show any clear effect toward increased intonation accuracy. Seven out of twelve participants responded saying that lessons with aural feedback were their favorite. Eight of twelve students thought combined aural and visual feedback would be the most helpful for individual practice. Both aural and visual feedback were praised for highlighting error. Aural feedback was more helpful at providing the correct target, more relevant to how a student self-corrects. Visual feedback was praised for being easier to identify how to correct. Only one student said they preferred having no feedback and two students said they were unlikely to use our intonation aids during practice.

Visual feedback often suffered from the need to compete for visual attention. Aural feedback highlighted error, but did not explicitly convey how to correct it and also took some time to get used to. Having both aural and visual feedback was praised for allowing use of aural feedback when visual attention was needed for other tasks while also having visual feedback available to clarify information from aural feedback.

We included a case study (section 3.3) demonstrating active use of aural feedback when a participant's intonation followed incorrect pitch settings. We also discussed issues relating to bias when using and answering questions related to feedback methods (section 3.2.1), how students largely trusted the intonation aids (section 3.2.7), and looked at the possibility but unlikelihood that positive remarks were because the experience was novel rather than valuable (section 4.1).

Along with tests for persistence, an interesting topic for future investigation is whether aggressively highlighting intonation error aurally is more effective for motivating students to correct intonation than the current more polite methods. Additionally, though we found evidence both methods of feedback were potentially useful, it would be necessary to test for persistence effects before either method of feedback was deemed truly valuable.

## Data Availability

Data including video is available on request to the authors, without undue reservation, to any qualified researcher. Due to the inclusion of children in the study, anonymous data sets and transcripts are publicly available on request, however video data can only be released if meeting proper ethical criteria.

## Ethics Statement

This study was carried out in accordance with the recommendations of Council of Queen Mary University of London Statement of Research Ethics Policy, Queen Mary Research Ethics Committee. The protocol was approved by the Queen Mary Research Ethics Committee. All subjects gave written informed consent in accordance with the Declaration of Helsinki.

## Author Contributions

LP acted as primary researcher, designing and conducting all research. Critical supervision of study design was provided by AM. The text of this article was written by LP with feedback and revision from AM.

### Conflict of Interest Statement

The authors declare that the research was conducted in the absence of any commercial or financial relationships that could be construed as a potential conflict of interest.

## Footnotes

^1^http://www.purelyviolin.com (Accessed: December 20, 2018).

^2^https://www.smartmusic.com (Accessed: December 20, 2018).

^3^We have not tested referenced commercial violin learning software. Information is based on our understanding of products as presented on their respective websites.

^4^https://trala.com (Accessed December 20, 2018).

^5^http://www.musicminusone.com (Accessed: December 20, 2018).

^6^https://gb.abrsm.org/en/exam-support/practice-tools-and-applications/scales-trainer/ (Accessed: December 20, 2018).

^7^https://www.playalongmusic.com/ (Accessed: December 20, 2018).

^8^We use the term pitch as it relates to frequency and utilize an equal-temperament scale. Conversion from Hz to linearized pitch is calculated using:

$$\begin{array}{r} f_{LI}\left(n\right)=-12log_{2}\left(\frac{440\text{Hz}}{f_{in}\left(n\right)}\right) \end{array}$$

^9^Preferences reported in Table 3 were based on final interviews only.

^10^The augmented violin's microphone captured the teacher's playing. Audio was then shifted based on the student's playing which would accidentally shift the teacher's playing so that it was no longer correct.

^11^The retuning software only snaps audio measured to be over a certain volume. When the level of the audio input signal was sufficiently low, pitch correction would turn off and on depending on momentary changes in volume. As a result, the greater the difference between the played pitch and the target pitch, the more noticeable the audio artifact became.
